# Supplementary material for: Correction of field instabilities in biomolecular solid-state NMR by simultaneous acquisition of a frequency reference
Source: Magn Reson (Gott). 2022 Feb 9;3(1):15–26. doi: 10.5194/mr-3-15-2022 (PMC10539777; doi:10.5194/mr-3-15-2022)
Supplement: The supplement related to this article is available online at: https://doi.org/10.5194/mr-3-15-2022-supplement. [file mr-3-15-supplement.pdf]

Supplement of Magn. Reson., 3, 15–26, 2022
https://doi.org/10.5194/mr-3-15-2022-supplement
© Author(s) 2022. CC BY 4.0 License.

Open Access
MAGNETIC RESONANCE

Supplement of

Correction of field instabilities in biomolecular solid-state NMR by simultaneous acquisition of a frequency reference

Václav Římal et al.

Correspondence to: Beat H. Meier (beme@ethz.ch)

The copyright of individual parts of the supplement might differ from the article licence.

S1 Supplementary figures

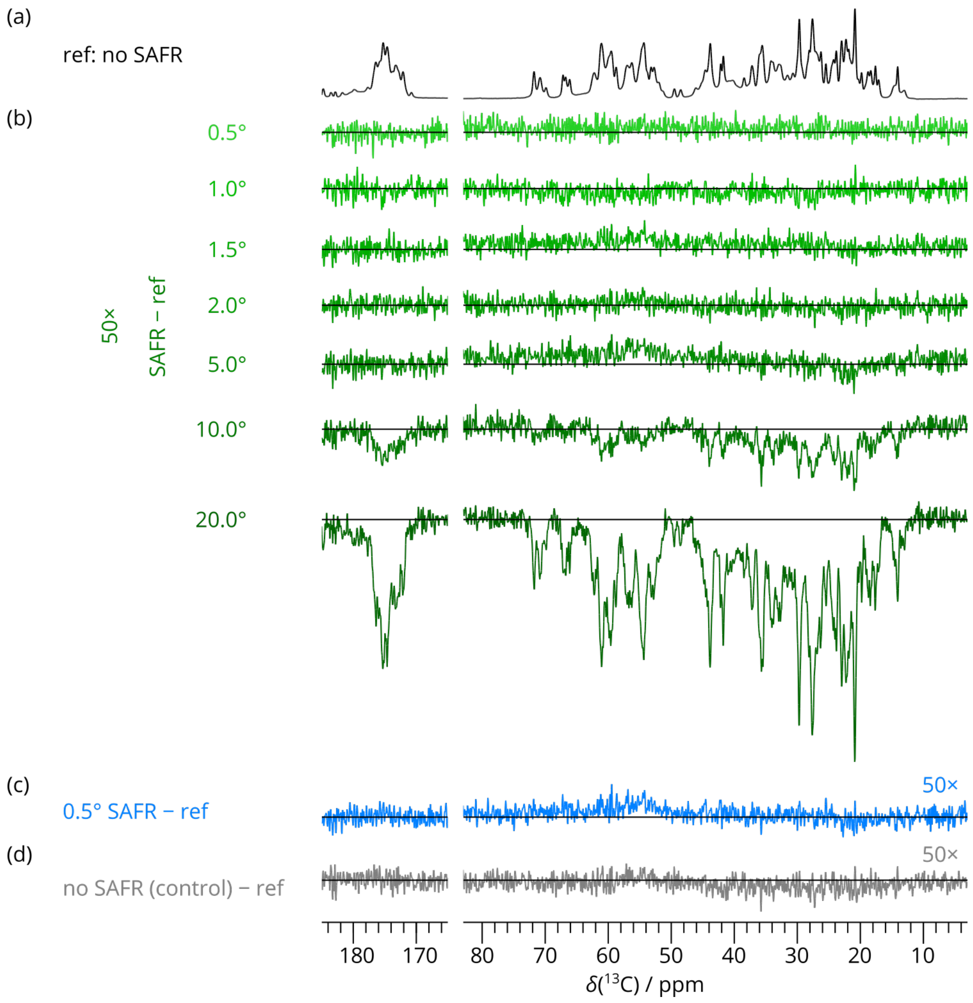

Figure S1: 1D ^{13}C DARR spectra of HETs(218–289) at various ^{1}H SAFR flip angle using pulse sequence in Fig. 2 (b) in the main text (1200 MHz). Acquisition parameters (128 scans, 64 dummy scans, relaxation delay 2.7 s), experimental time (8 min 49 s), and processing parameters (no apodization, common phase correction, automatic fifth-order polynomial baseline correction) of each spectrum were otherwise the same. All spectra were run immediately one after another from top to bottom. (a) A reference spectrum with no SAFR pulse. (b) Difference spectra between 1D ^{13}C DARR spectra after ^{1}H SAFR pulse with flip angle varying from 0.5° to 20.0° and the reference spectrum in (a). (c) Difference spectrum between another 1D DARR after 0.5° SAFR and the reference. (d) Difference between a control spectrum with identical parameters as in (a) and the reference spectrum in (a). The difference spectra in (b), (c), and (d) are scaled 50 times up relative to (a).

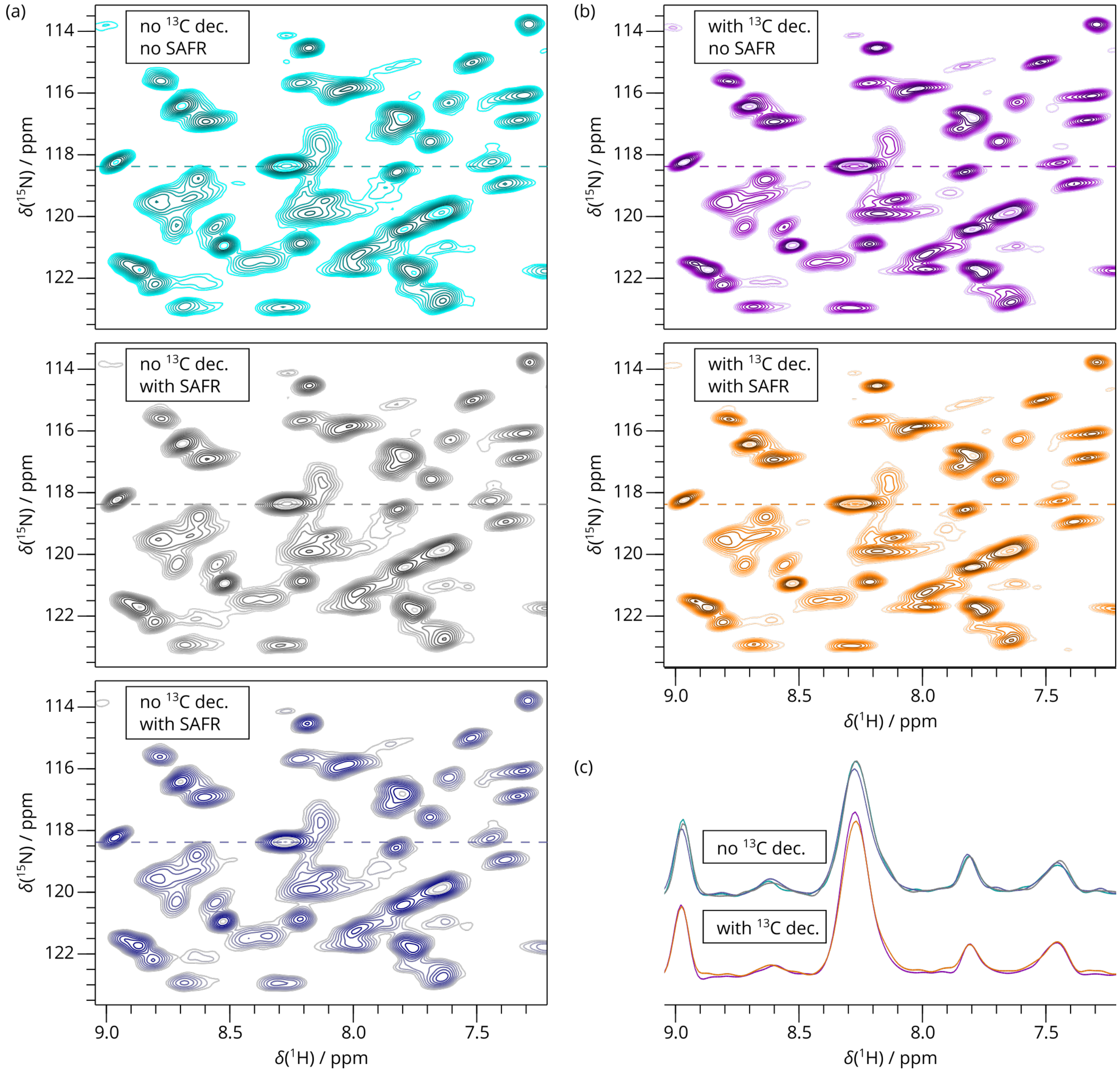

Figure S2: Comparison of 2D hNH experiments of dASC recorded with various options of the pulse program safr-hNH (850 MHz). Acquisition parameters (400 t_1 increments, 16 scans per FID, SAFR flip angle 0.5°) and experimental time (2 h 35 min 8 s) of each spectrum were otherwise the same. No drift correction was applied. (a) The effect of SAFR while ^{13}C decoupling was turned off: a spectrum with SAFR switched off (top) and two spectra with SAFR on (control 1 and control 2 shown in Fig. S11 as well) were recorded in order to show possible differences caused by SAFR as well as random changes. (b) ^{13}C decoupling turned on: SAFR off (top) and on (bottom). (c) 1D traces along the horizontal dashed lines in (a) and (b).

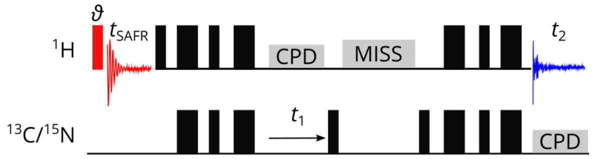

Figure S3: Pulse program for ^{1}H-detected SAFR (red, flip angle ϑ) before a ^{1}H-detected 2D hCH or hNH correlation experiment with refocused INEPT transfers. Thin and thick filled black rectangles represent 90-degree and 180-degree pulses, respectively, and grey blocks show composite-pulse decoupling (CPD) and MISSISSIPPI water suppression (MISS). Optionally, decoupling of the third nucleus is turned on during t_1 and t_2.

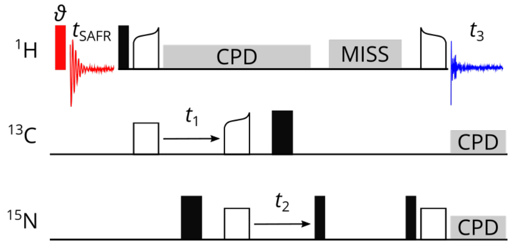

Figure S4: Pulse program for ^{1}H-detected SAFR (red, flip angle ϑ) before a CP-based ^{1}H-detected 3D hCNH correlation experiment. Thin and thick filled black rectangles represent 90-degree and 180-degree pulses, respectively, empty rectangles and curved shapes are CP transfers and grey blocks show composite-pulse decoupling (CPD) and MISSISSIPPI water suppression (MISS).

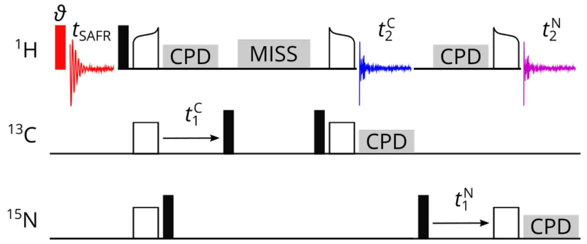

Figure S5: Pulse program for ^{1}H-detected SAFR (red, flip angle ϑ) before a dual-acquisition MAS (DUMAS) ^{1}H-detected 2D hCH (blue acquisition) and hNH (violet acquisition) correlation experiment with CP transfers. The number of increments of the indirect evolution periods t_1^C and t_1^N for the hCH and hNH experiments, respectively, is the same, but their maximum evolution times (hence resolutions) are set independently. Filled black rectangles represent 90-degree pulses, empty rectangles and curved shapes are CP or simultaneous-CP transfers and grey blocks show composite-pulse decoupling (CPD) and MISSISSIPPI water suppression (MISS).

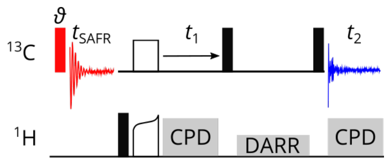

Figure S6: Pulse program for ^{13}C-detected SAFR (red, flip angle ϑ) before a 2D ^{13}C DARR. A single channel is used for both acquisitions. Although the initial ^{1}H–^{13}C CP transfer in the main experiment prohibits CP and ^{1}H decoupling during SAFR, because it would saturate protons before the main experiment starts, a 90-degree SAFR pulse (ϑ = 90°) can be employed to gain a stronger signal. Filled black rectangles represent 90-degree pulses, empty rectangle and curved shape is the CP transfer and grey blocks show high-power composite-pulse decoupling (CPD) and the DARR.

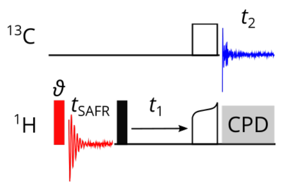

Figure S7: Pulse program for ^{1}H-detected SAFR (red, flip angle ϑ) before a ^{13}C-detected 2D ^{1}H–^{13}C correlation. Filled black rectangle represents a 90-degree pulse, empty rectangle and curved shape is the CP transfer and the grey block shows high-power composite-pulse decoupling (CPD).

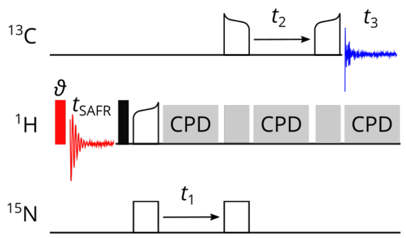

Figure S8: Pulse program for ^{1}H-detected SAFR (red, flip angle ϑ) before a CP-based ^{13}C-detected 3D hNCC correlation experiment. Filled black rectangles represent 90-degree pulses, empty rectangles and curved shapes are CP and DREAM transfers, and grey blocks show high-power composite-pulse decoupling (CPD) and decoupling by a continuous-wave irradiation avoiding ^{1}H recoupling conditions (without text).

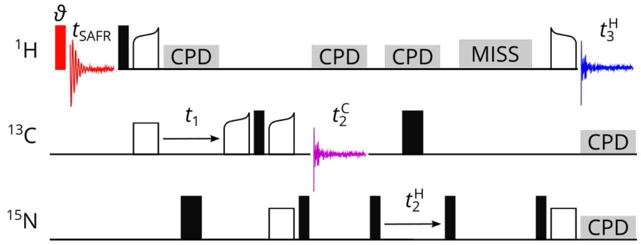

Figure S9: Pulse program for ^{1}H-detected SAFR (red, flip angle ϑ) before a ^{1}H-detected 3D hCcNH correlation experiment with CP and ^{13}C DREAM transfers (blue acquisition) with an additional ^{13}C detection of 2D hCC correlation exploiting orphan ^{13}C polarization after CP to ^{15}N (violet acquisition). Superscripts in the evolution periods mark whther they belong to the ^{13}C- or the ^{1}H-detected spectrum. The ^{13}C-detected FID is stored after every complete cycle of t_2^H increments, yielding more scans per FID in the 2D than in the 3D. Thin and thick filled black rectangles represent 90-degree and 180-degree pulses, respectively, empty rectangles and curved shapes are CP or DREAM transfers and grey blocks show composite-pulse decoupling (CPD) and MISSISSIPPI water suppression (MISS).

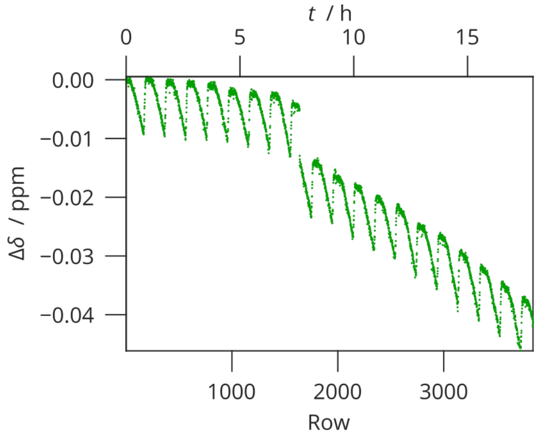

Figure S10: Natural field drift of the 1200 MHz magnet (linear hardware correction turned on with a fixed drift value) observed by ^{13}C CP on adamantane using a pseudo-2D pulse sequence. Positions of the methylene peak (38.5 ppm) were determined by safrcorr procedure. Non-linear field drift can be observed, as well as periodic oscillations on top of it.

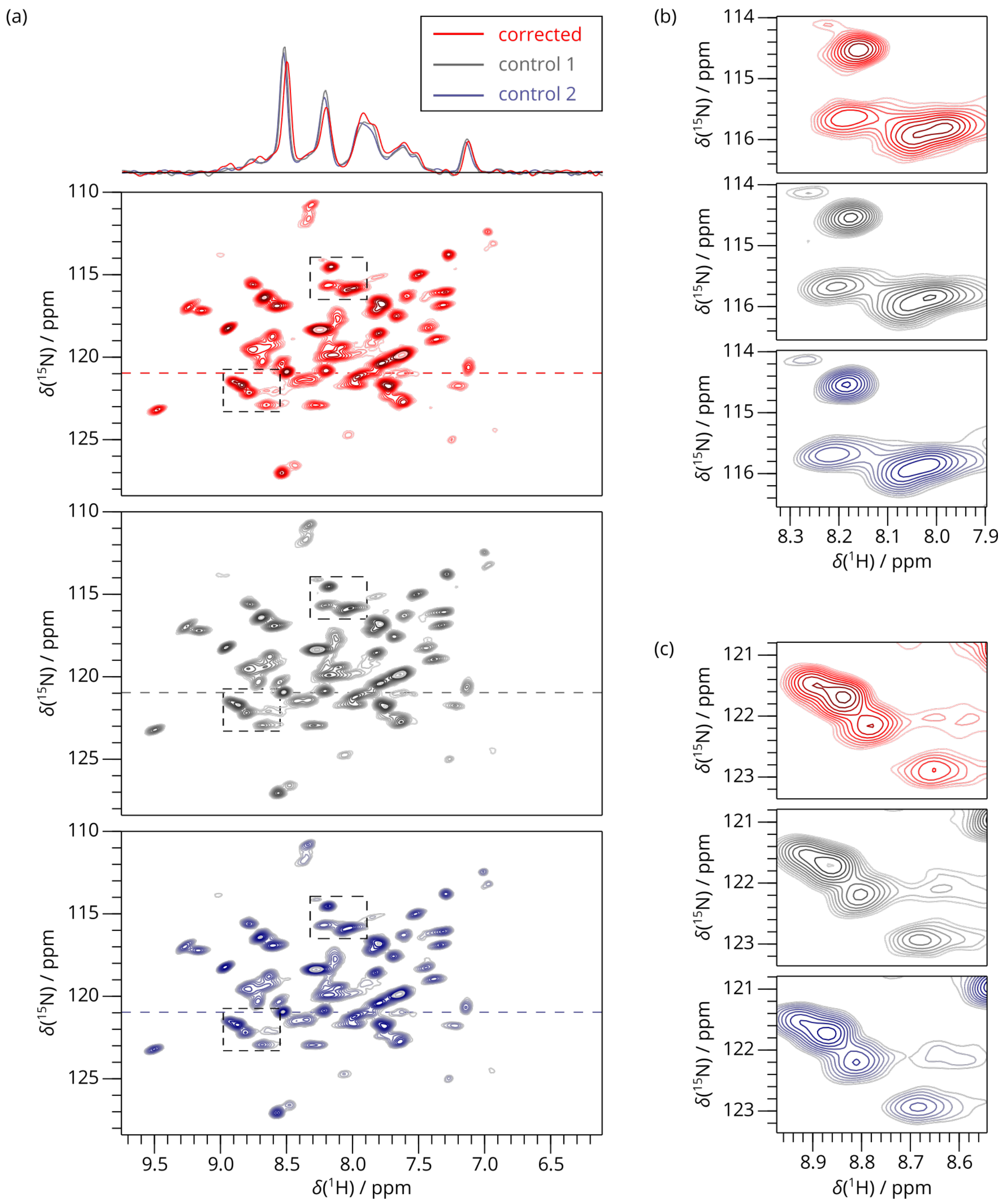

Figure S11: 2D SAFR–hNH of dASC 36 min after the start of cooling down with drift correction (Fig. 4 in the main text) and control experiments run at stable conditions with no correction applied (850 MHz). (a) Drift-corrected spectrum acquired during cooling down (red) and two control experiments (grey and dark blue). 1D traces along the horizontal dashed lines are shown at the top. (b), (c) Two selected details at positions indicated by the dashed rectangles in (a).

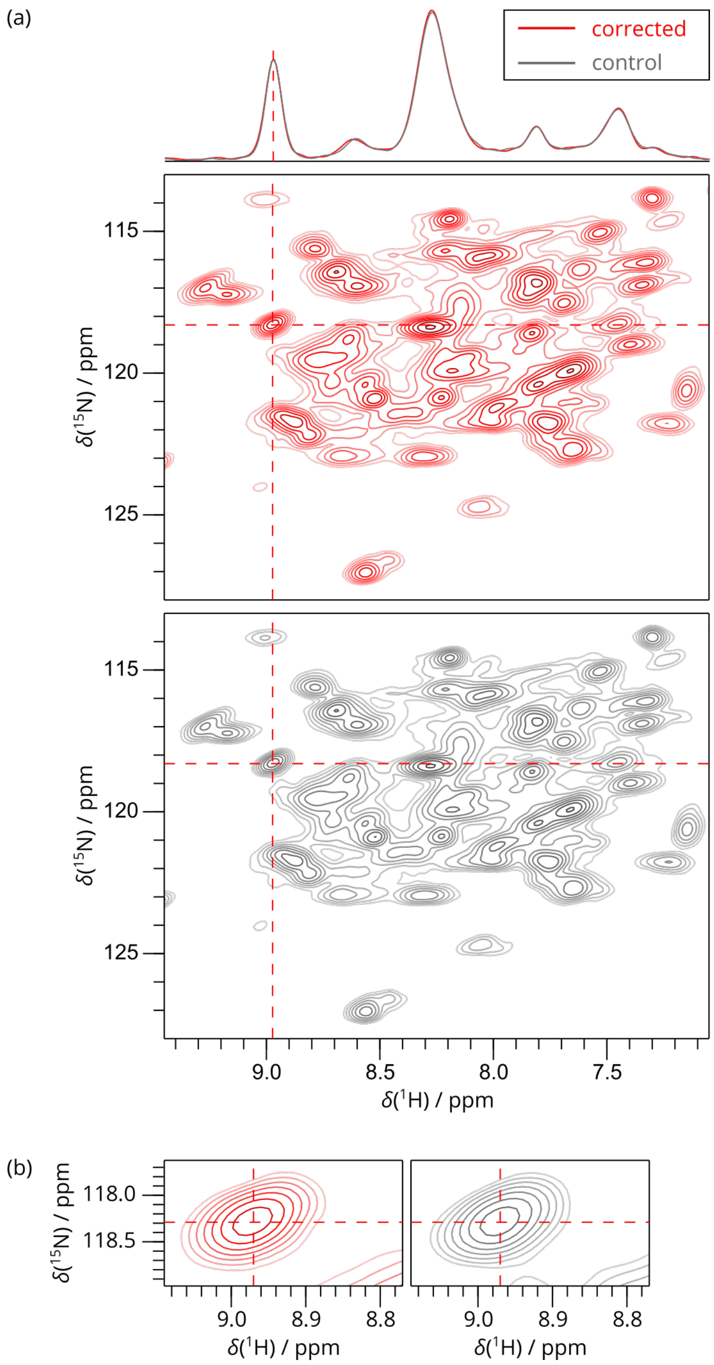

Figure S12: 2D SAFR–hNH of dASC during helium fill with drift correction (Fig. 5 in the main text) and a control experiment during a negligible field drift (12 Hz in ^{1}H) with no correction applied (850 MHz). (a) Drift-corrected spectrum during helium fill (sum of four datasets with 8 scans each, red) and a control experiment (one spectrum with 32 scans, grey). 1D traces along the horizontal dashed lines are shown at the top. (b) Selected peak, indicated by dashed lines in (a), in detail.

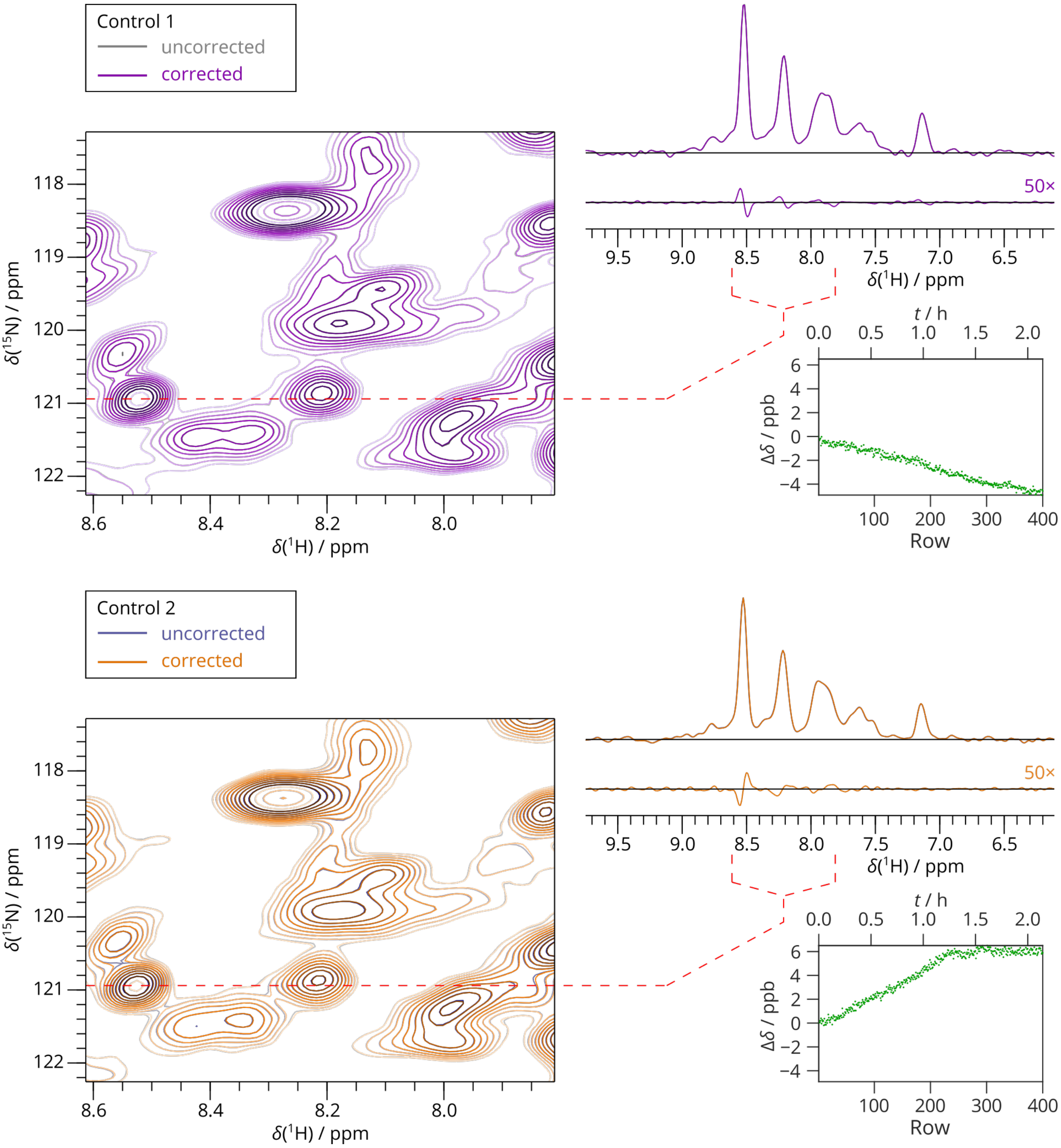

Figure S13: Control 2D SAFR–hNH experiments of dASC as in Fig. S11, but in overlay with the same spectra after the drift correction. Almost no differences are visible. The 1D traces through the indicated ^{15}N frequency are shown together with the difference spectra between the corrected and uncorrected data (magnified 50-fold). The evolutions of the proton frequency drift are shown in parts per billion (ppb).

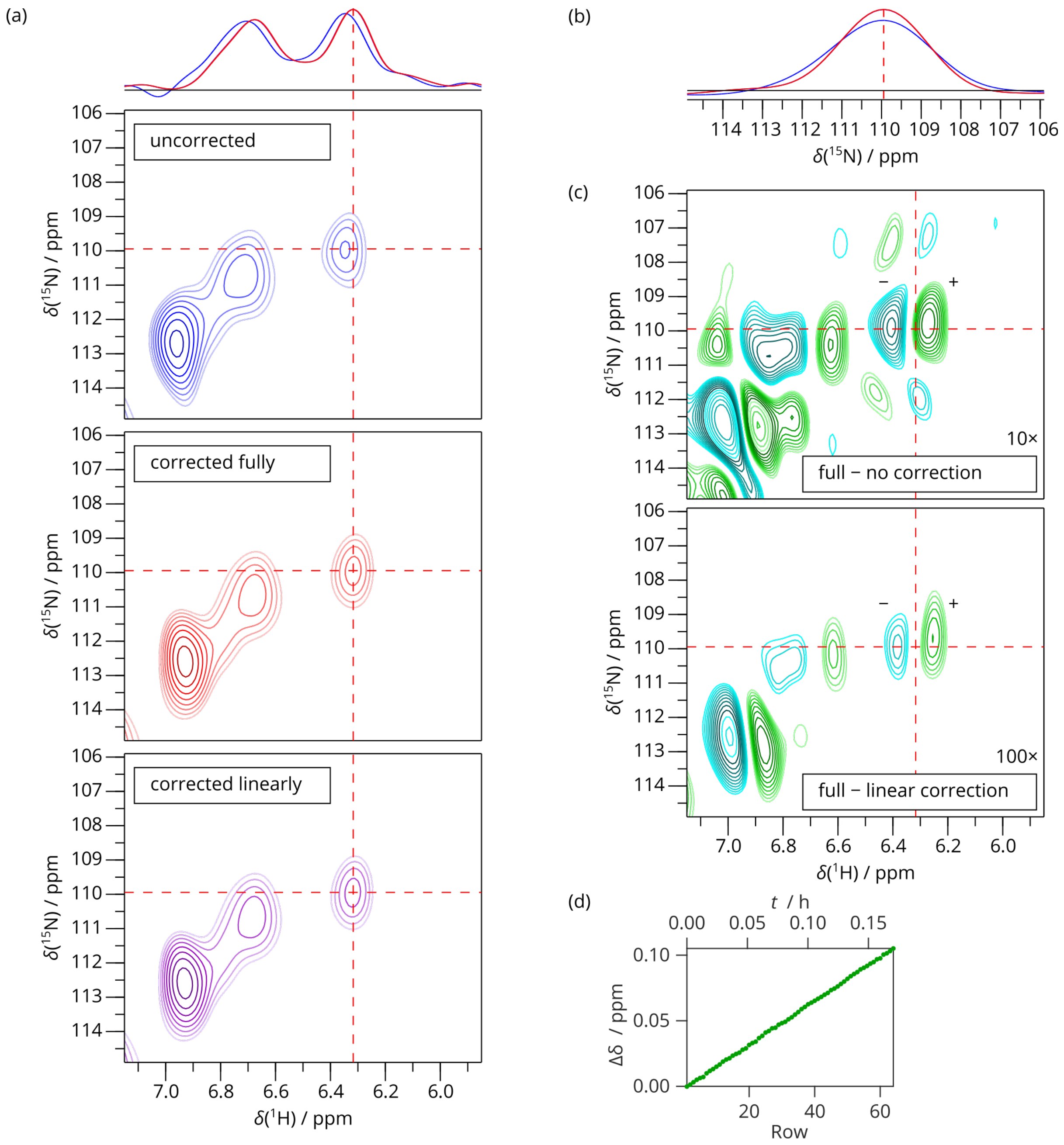

Figure S14: 2D SAFR-hNH of ASC with a linear field drift −0.638 ppm/h (850 MHz). (a) A detail of the spectrum after no correction, linear correction using lindriftcomp (Najbauer and Andreas, 2019), and full drift correction using safrcorr. 1D traces along the dashed red lines (^{1}H dimension) are shown at the top. (b) 1D traces of the spectra in (a) along the dashed red lines (^{15}N dimension). (c) The differences of the spectra in (a) with the same contour levels, but scaled 10- and 100-fold as indicated. (d) The frequency drift as measured by ^{1}H SAFR and calculated from the H_2O peak maxima using safrcorr.

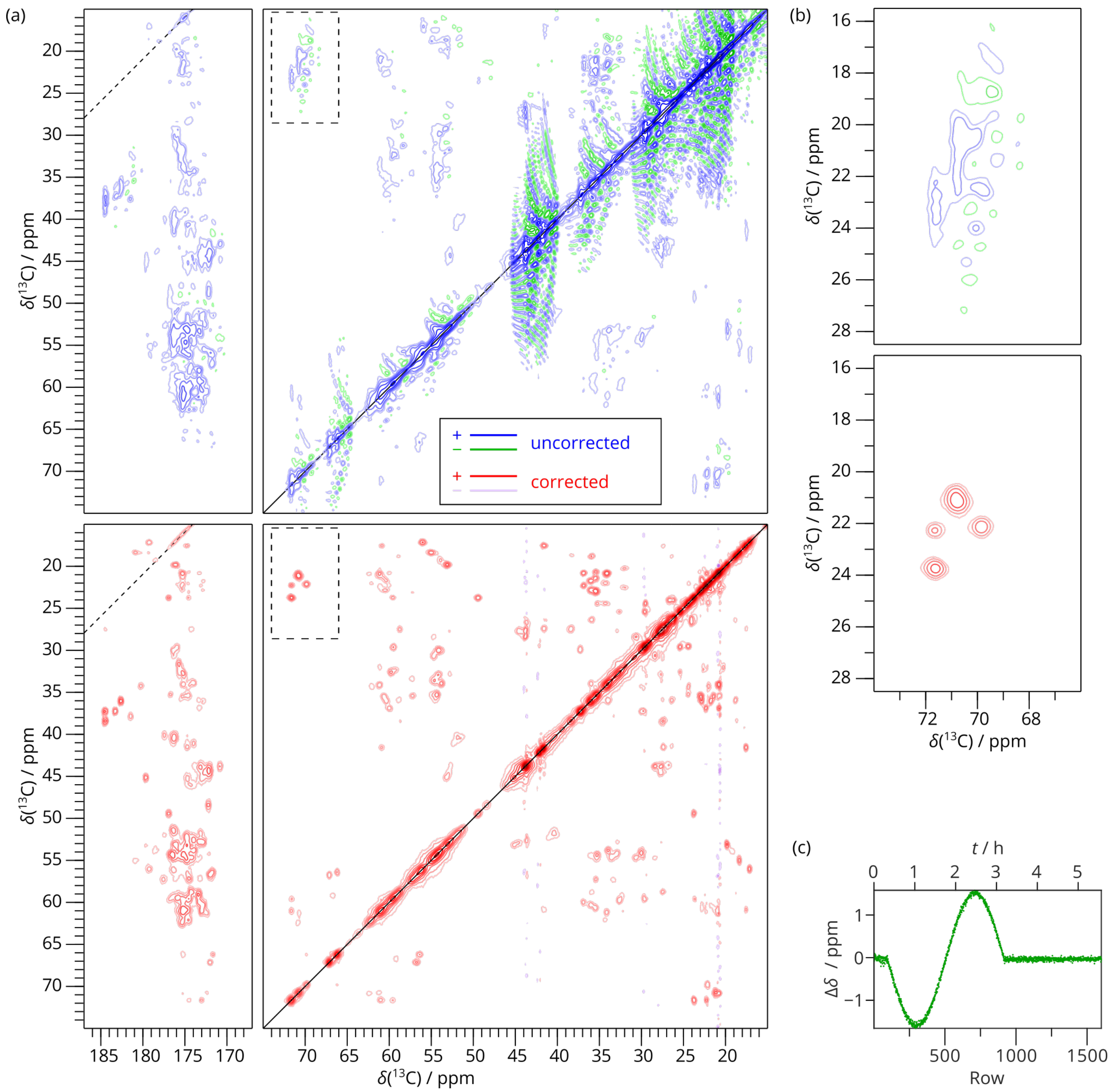

Figure S15: 2D ^{13}C-DARR with ^{13}C-SAFR (pulse program in Fig. S6, SAFR pulse 10°) of HET-s(218–289) during a sine drift (850 MHz). (a) The 2D spectrum before (top, blue) and after the drift correction (bottom, red). The black diagonal line indicates the spectral diagonal and the dashed line parallel to it marks the position of spinning side bands. (b) Detail of the region indicated by dashed rectangles in (a). (c) The frequency evolution in terms of the chemical shift difference of the carbonyl resonance at 173 ppm measured by ^{13}C-SAFR.

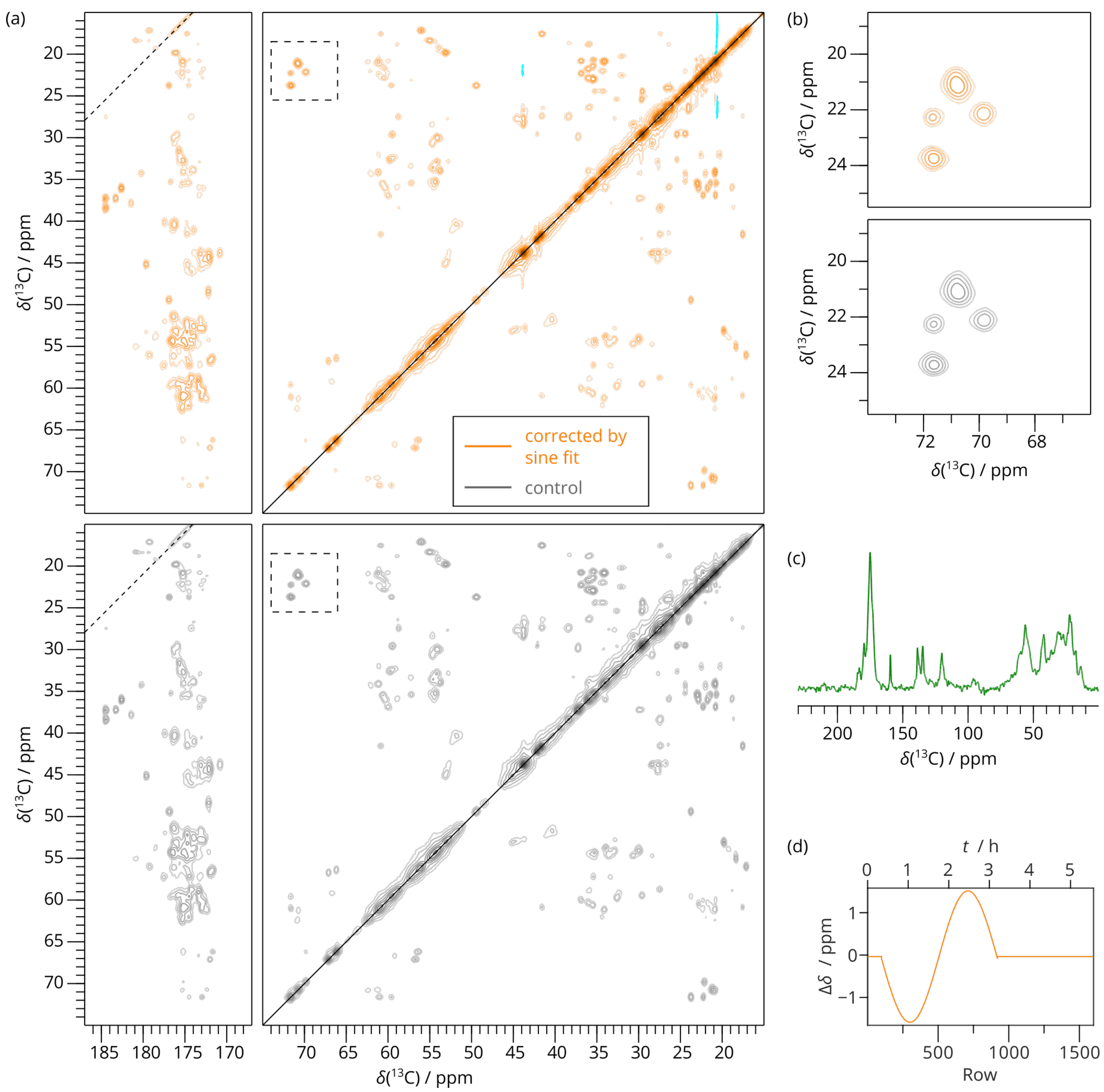

Figure S16: 2D ^{13}C DARR with ^{13}C-SAFR of HET-s(218–289) during a sinusoidal field change as in Fig. S15, but with correction according to a function fitted to the measured drift and a control experiment run at stable conditions with no correction applied (850 MHz). (a) Drift-corrected spectrum acquired during sine drift and a control. (b) Details of the region indicated by dashed rectangles in (a). (c) A ^{13}C SAFR spectrum acquired along the spectrum in (a). (d) The fit of the frequency evolution by a sine function and a constant value outside the sinusoidal part used for correction giving the spectrum in (a).

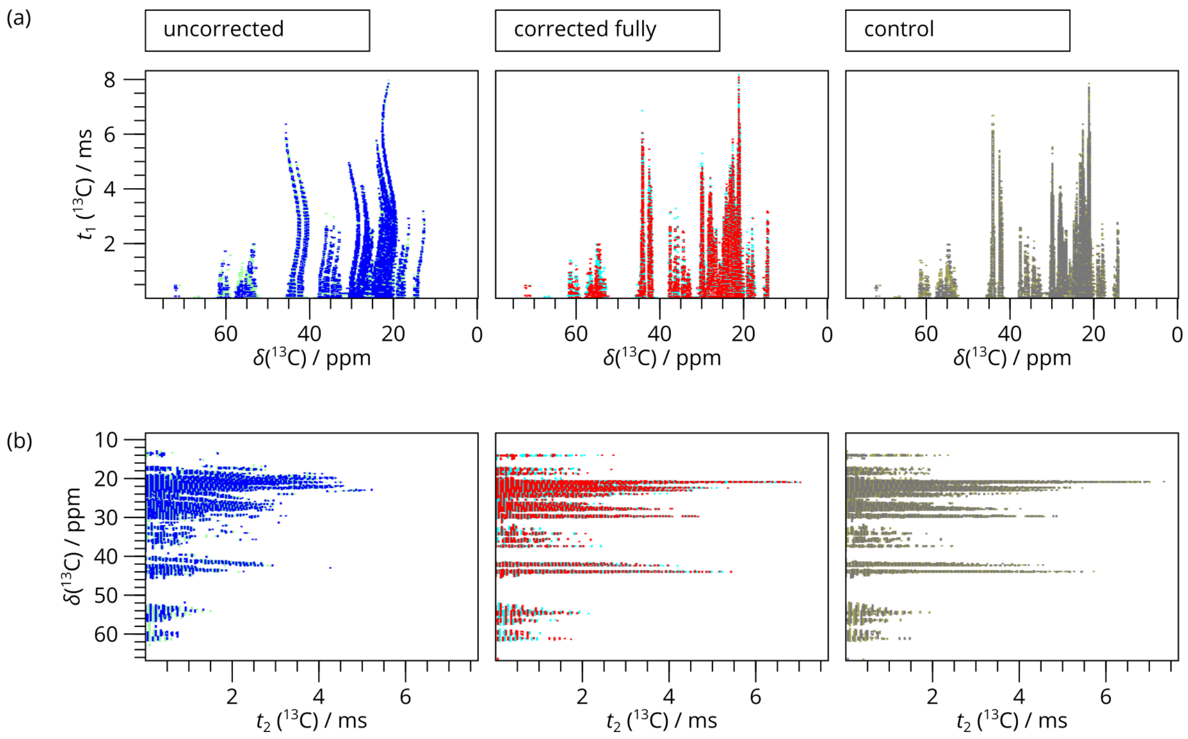

Figure S17: Partially Fourier-transformed 2D ^{13}C DARR with ^{13}C-SAFR of HET-s(218–289) during a sinusoidal field change before and after drift correction and a control experiment run at a stable field with no correction (fully transformed spectra shown in Fig. S15 and S16). (a) FT done along direct dimension only. (b) FT done along the indirect dimension only.

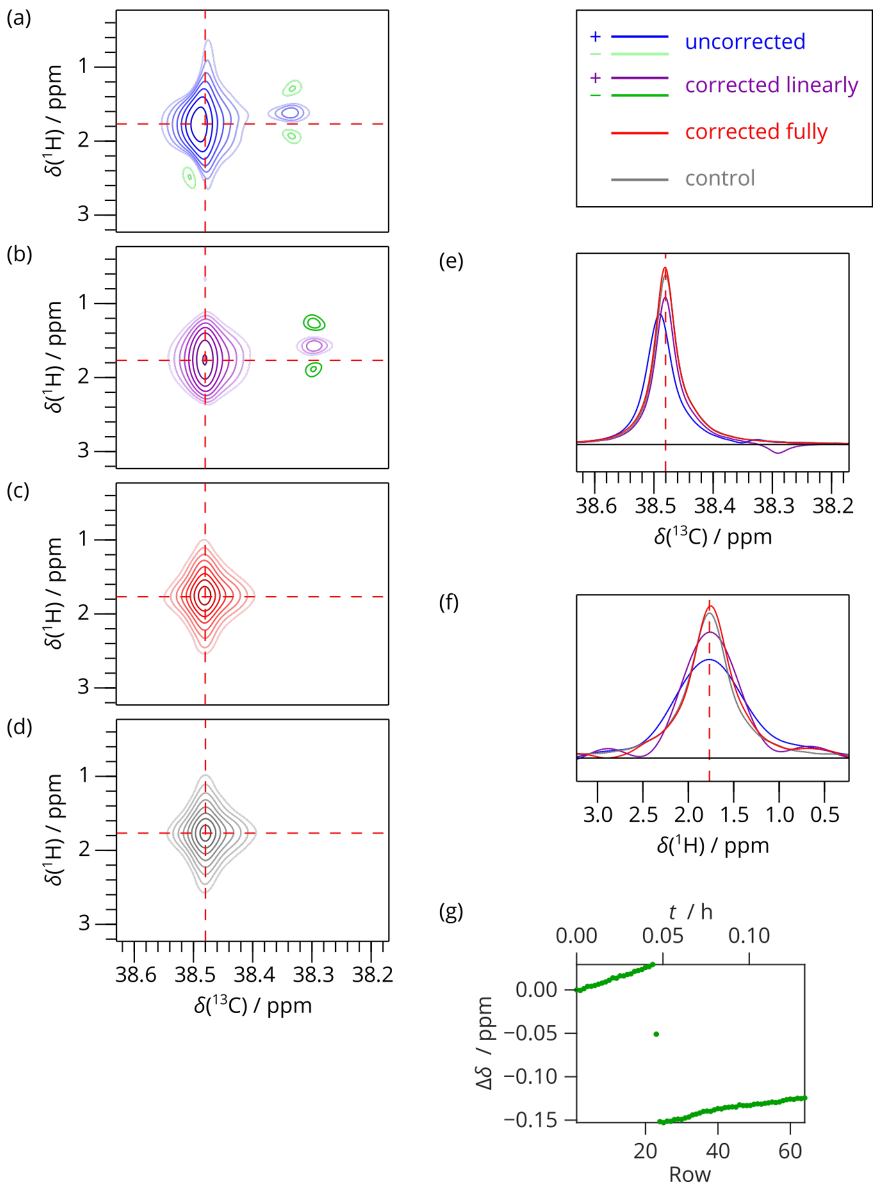

Figure S18: 2D SAFR–HC (^{13}C detection of the main 2D with ^{1}H-detected SAFR, pulse program in Fig. S7) of ^{13}C-adamantane (1200 MHz) during deliberate field manipulations by Z^0 shim current. Exponential line broadening by 2 Hz and 100 Hz was applied in the ^{13}C (direct) and ^{1}H (indirect) dimensions, respectively, in order to preserve the Lorentzian line shapes, in contrast to squared sine-bell apodization used in other spectra in this work. (a) The methylene peak without any correction. (b) Spectrum from (a) with linear drift correction by −25 Hz (−0.08 ppm, giving the best result) using lindriftcomp (Najbauer and Andreas, 2019). (c) Fully corrected spectrum using safrcorr, the reference being the adamantane ^{1}H resonance acquired by SAFR. (d) Control spectrum measured without SAFR during a naturally drifting field with the linear hardware correction turned on. (e, f) ^{13}C and ^{1}H 1D slices, respectively, through the spectra in (a–d) along the dashed red lines. (g) The frequency drift as measured by ^{1}H SAFR and calculated from the peak maxima using safrcorr. A fast jump in the field value was made during acquisition of row 23. An average shift appears in the plot, because the frequency change was smaller than the ^{1}H linewidth.

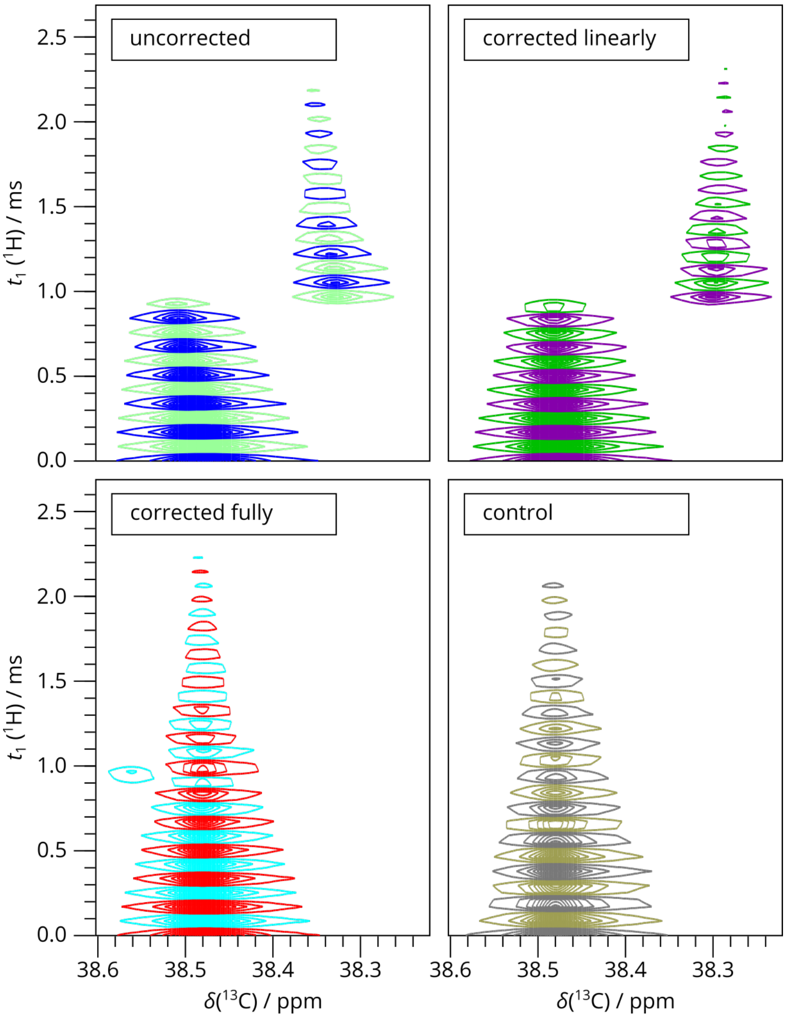

Figure S19: 2D SAFR–HC of ^{13}C-adamantane during deliberate field manipulations as in Fig. S18 with Fourier transform in ^{13}C (direct) dimension only. The alternating sign (different colors) corresponds to the phase changes in the States–TPPI phase cycle. The evolution of the methylene resonance is shown after no correction, linear correction by lindriftcomp (Najbauer and Andreas, 2019), full correction by safrcorr, and an uncorrected control experiment. The fast jump in the field value was made during acquisition of row 32 (4 scans). The frequency change was higher than the ^{13}C linewidth, which caused a doubling of the spectrum that cannot be fully compensated in this particular row.

S2 Supplementary tables

Table S1: All 2D and 3D pulse programs for Bruker Topspin forming part of this publication. All include ^{1}H-detected SAFR, except for safrC-DARR which employs a ^{13}C-SAFR instead

^{1}H detected		^{13}C-detected	
2D	3D	2D	3D
`safr-hCH`	`safr-hCNH`	`safr-HC`	`safr-hNCC`
`safr-hCH-rINEPT`	`safr-hCcNH`	`safr-DARR`	
`safr-hNH`	`safr-hCc-Cdet-NH`	`safrC-DARR`	
`safr-hNH-rINEPT`			
`safr-hCH-hNH-DUMAS`			

Table S2: Acquisition and processing parameters of the 2D spectra

	**dASC** cooling hNH	**dASC** He fill hNH	**Rpo4/7*** N_2 fill hCH	**dASC** options hNH	**ASC** linear drift hNH	**HET-s(218–289)** ^{13}C DARR[a]	**Adamantane** HC
Figures	4, S11, S13	5, S12	7	S2	S14	S15, S16, S17	S18, S19
NS	16	8[b]	8	16	8	8	4
D1 / s	1.0	1.0	1.2	1.0	1.0	1.5	1.5
Duration / h	2.15	1.39	3.24	2.15	0.171	5.56	0.132
TD (f_2)	4096	2048	16384	4096	2048	4096	32768
TD (f_1)	400	512	1024	400	64	2048	64
AQ (t_2) / ms	51.61	60.21	81.92	51.61	25.80	34.95	163.84
AQ (t_1) / ms	58.04	42.44	21.19	58.04	5.31	17.82	2.69
SW (f_2) / ppm	46.67	20.00	83.27	46.67	46.67	274.12	331.15
SW (f_1) / ppm	39.99	70.00	79.99	39.99	70.00	268.86	9.91
WDW	QSINE	QSINE	QSINE	QSINE	QSINE	QSINE	EM[c]
SSB / LB	2.5	2	5	2.5	2	2	2 Hz / 100 Hz
SI (f_2)	4096	2048	4096	4096	4096	4096	131072
SI (f_1)	1024	1024	2048	1024	512	4096	512
TD_{eff} (f_2)	2048	1024	1024	2048	1024	4096	32768
TD_{eff} (f_1)	400	512	1024	400	64	1600	64

[a]Differently from all other spectra, DARR was acquired in TPPI mode and converted to States–TPPI by `convdta`, inverse Fourier transform by `xtrfp` followed by `genser` and modification of the acquisition status parameter `FnMODE` in Topspin
[b]Values for the four individual blocks as in Fig. 4 are shown; the control experiment had NS = 32
[c]Exponential line broadening shown for direct and indirect dimension, respectively

Table S3: Acquisition and processing parameters of the 3D spectrum

	**dASC**
	hCANH
Figure	6
NS	8
D1 / s	1.5
Duration / h	70.6
TD (f_3)	3072
TD(f_2)	122
TD(f_1)	148
AQ(t_3) / ms	64.51
AQ(t_2) / ms	7.16
AQ(t_1) / ms	3.57
SW(f_3) / ppm	19.82
SW(f_2) / ppm	70.00
SW(f_1) / ppm	70.00
WDW	QSINE
SSB	2
SI (f_3)	4096
SI (f_2)	64
SI (f_1)	128
TD_{eff} (f_3)	1024
TD_{eff} (f_2)	122
TD_{eff} (f_1)	148

S3 Description and usage of pulse and AU programs

S3.1 Pulse programs

- All the pulse programs listed in Table S1 are available online (Římal, 2022).
- The pulse programs are compatible with and have been tested on Bruker Avance III, III+, and NEO with Bruker Topspin 3 or 4.
- Extensive comments and hints for preparation of the experiment and processing are present in the pulse programs.
- Acquisition parameters are explained in the pulse programs (available also in Topspin after `ased` command).
- Several control options can be used in the `ZGOPTNS` acquisition parameter:
  - `-Dnosafr`: turns off the SAFR block (no SAFR pulse, no additional acquisition);
  - `-Donedim`: makes the pulse program 1D (SAFR pulse present, but not its acquisition);
  - `-DCdec` for 2D hNH or `-DNdec` for 2D hCH: turn on decoupling on third channel.
- 1D variant of every pulse program (except for `safr-hCH-hNH-DUMAS`) is supplied as well, using the directive `#define onedim` and inclusion of the main pulse program.
- Consistency of the numbering of pulses and delays within all ^{1}H-detected pulse programs allows a stepwise optimization of a pulse sequence (e. g. safr-hNH ▸ safr-hCH ▸ safr-hCNH for a 3D hCANH or hCONH experiment).
- Pulse programs are intentionally written relatively simple and instructive on the use of SAFR.
- 2D and 3D pulse programs are available, but extension to more dimensions is straightforward.
- SAFR pulse is always `P18` at `PLW18` power (18 = R as reference):

  ```
  (p18 pl18 ph18):H
  ```

  and its FID is acquired by the command `goscnp`.
- Homonuclear SAFR: `st0` and `st` are used to store the FIDs into separate memory buffers, one `ser` file is produced.
- Heteronuclear SAFR: two receivers are active, two datasets (expnos) are used, two `ser` files are produced. The heteronuclear SAFR needs a hardware support of multiple receivers, implemented routinely by Bruker since Avance NEO.
- Because of its limited compatibility with multiple acquisitions during one cycle of a pulse program, the `mc` macro is replaced by `wr` with a fixed States–TPPI loop scheme:
  - auxiliary delays `acqTime` (and `acq2Time` for heteronuclear SAFR), `incrTime`, and `writeTime` as well as a counter `td1States` (and `td2States` for 3D) are defined in order to facilitate and generalize the code;

- for example, the mc statement

  ```
  1m mc #0 to 2 F1PH(ip9, id0)
  ```

  in a 2D experiment with homonuclear SAFR is replaced by

  ```
  writeTime wr #0 if #0 zd ip9
  lo to 3 times 2
  incrTime id0
  lo to 4 times td1States
  ```

  and the labels in the beginning of the pulse program are modified accordingly with appropriate delays that preserve the duration of each cycle;
- a pulse program with heteronuclear SAFR, such as 2D ^{13}C DARR, uses such commands to store the data from the two receiver on the disk:

  ```
  writeTime wr1 #0 if1 #0 zd1 ip5
  writeTime wr2 #1 if2 #1 zd2
  ```

- as a consequence of avoiding the `mc` clause, it is not enough to switch the dimensionality of an experiment by the `1, 2,` … button in `eda`, but adding or removing the `-Donedim` option is also necessary;
- the absence of the `mc` macro makes implementation of NUS or other alternative schemes challenging.

• While D1 is still the duration of the repetition delay set by the user, D11 is used and calculated internally in the pulse programs. This conserves the overall timing regardless if SAFR is used or not. It also takes the delays introduced due to fixed States–TPPI cycle into account. In a homonuclear SAFR 2D, D11 is calculated as follows:

```
#ifdef nosafr
  "d11=d1-writeTime-incrTime"
#else
  "d11=d1-writeTime-incrTime-p18-acqTime-incrTime-d12"
#endif
```

S3.2 Preparation of experiments with SAFR – a typical workflow

We recommended here a way of preparing a new experiment using exclusively the pulse programs with SAFR:

1. optimizing all the pulse and delay parameters using a desired pulse sequence from Table S1 (or a sequential usage of several pulse programs, from a simple one to more complex ones) with `-Donedim` specified in `ZGOPTNS` (or, equivalently, using a `<name>-1D` pulse program), possibly with `-Dnosafr` as well;
2. SAFR pulse is set by `P18` and `PLW18` (a lower power than for a hard pulse is advisable);
3. experiment is switched to 2D or 3D in `AcquPars` and the `-Donedim` option is removed (or the pulse program is switched to the name without the `-1D` suffix);
4. parameters of the indirect dimensions are set as for normal experiment;
5. for an experiment with homonuclear SAFR, one of the indirect `TD` values needs to be doubled (described in the header of every pulse program);

6. in the heteronuclear variant, a second dataset must be prepared for the second receiver, e. g. using the button `C` in the `AcquPars` window.

S3.3 Pre-processing and drift correction

The recommended steps after acquisition are as follows:

1. spectral axis calibration by standard means according to the beginning of the experiment;
2. for homonuclear case, splitting of the spectrum into new datasets by Topspin native AU program `split` is needed (`split` creates experiments with increasing numbers, usage of the first one – the reference spectrum with SAFR – ending with “`00`” is recommended);
3. processing of the reference experiment in a pseudo-2D way (`xf2` for 2D or `xf2 nd2d` for 3D) and a proper phasing by the user;
4. running `safrcorr` on the main experiment (i.e., ending with “`01`” if the recommendation in step 2 is followed);
5. standard processing (apodization, Fourier transform, etc.) of the corrected experiment under the new number.

S3.4 Description of the AU program `safrcorr`

- The program is available online (Římal, 2021).
- The user is prompted for several inputs, but the default values could usually be used:
  - “Intermediate expno for storing ser with ‘int’ format”: if `double` data type of the `ser` file is detected (normally on Avance NEO), a conversion to `int` is done by `sertoint` and a new `expno` is required;
  - “Expno with the reference pseudo-2D SAFR spectrum”: the `expno` of the reference spectrum with SAFR;
  - “Procno with reference pseudo-2D after xf2”: for 3D experiments, the processing number (`procno`) of the reference spectrum (for 2D experiments, `procno` 1 is always used);
  - “The initial shift in Hz”: the initial value of field drift: this can be used to process a row of identical experiments before they are added together, otherwise zero should be the input; positive value shifts the entire spectrum to higher frequencies; the units correspond to the direct dimension of the main experiment in Hz;
  - “New experiment number”: the desired new `expno` for the corrected data.
- The core of the drift correction includes:
  - reading the processed data (file `2rr`) of the reference experiment with SAFR;
  - finding global maximum and its parabolic interpolation from three adjacent data points (Press et al., 2016) of every row of SAFR data (equivalent result to the `pp` command in Topspin applied on every row, but much faster);
  - reading the raw data (`ser`) and the necessary parameters of the main 2D or 3D experiment;

- shifting the phases of the acquired complex data in the time domain according to Eqs. (3) and (7) in the main text using the frequency change relative to the first row of SAFR (and the initial shift, if specified by the user);
- storing the corrected data as a new `ser` under the selected `expno`;

- The output of `safrcorr` consists of:
  - the corrected data in the new `expno` (no processing done);
  - the peak positions and the field drift determined from SAFR displayed on the screen and stored into the file `drift.txt` in the processed data directory of the main `expno`;
  - a standardized Topspin audit file `audita.txt` in the directory of the new `expno`;
  - several status messages in Topspin that indicate key points of the program run.
- Errors are handled as follows:
  - `safrcorr` quits, if the data are not 2D or 3D, if the sampling in any indirect dimension is not planes, if a 3D data with echo–antiecho mode is encountered, if a call to a built-in macro or another AU program exits with an error, if the Fourier-transformed reference spectrum is missing, or if the total number of FIDs exceeds an internal limit of 65536;
  - if QSEQ, TPPI, or undefined acquisition schemes are encountered, a question is displayed whether to proceed;
  - a question whether to continue or abort is asked, if the number of the rows of the processed reference spectrum with SAFR is smaller than the total number of acquired FIDs (`SI < TD` in the indirect dimension);
  - a warning is displayed, if a change in the reference frequency between adjacent rows in the reference spectrum with SAFR exceeds 10 Hz;
  - if the new `expno` already exists, a question is shown whether to overwrite, enter another expno, or abort.
- If the reference data use a submatrix and not the sequential storage, `safrcorr` does the appropriate reprocessing with `XDIM = SI.`
- Some pulse programs (but none of those published along this article) use such a phase changes connected to increments of t_{indir} that require frequency reversal in the corresponding indirect dimension. For every dimension with the `REVERSE` parameter equal to `TRUE`, this is handled by `safrcorr` before the drift correction by complex conjugation of the time-domain data. In the new experiment, the `REVERSE` parameters are all set to `FALSE`., because the reversal of frequency is already done by `safrcorr`.
- The program `safrcorr` uses parts of `lindriftcomp` by Najbauer and Andreas (2019).

S3.5 Auxiliary programs for sine drift

The artificial sinusoidal drift of the magnetic field during the DARR experiment on HETs(218–289) in Figures S15, S16, and S17 was created by a dedicated AU program. It introduced a single 10000 s long period (shorter than the

entire experiment which lasted 5 h 34 min) of a sine function with amplitude 250 field units (Hz of ^{2}D). The program calculated the desired field value every 10 s and stored it into the parameter `FIELD` of the BSMS unit by the macro `PutBsmsFloatValue`. This program was started during row 196 of the DARR experiment.

The DARR spectrum in Fig. S16 was corrected using the fitted sine function by another auxiliary AU program. It worked in the same way as `safrcorr`, except that the drift was not read from the reference experiment, but fixed to the result of the fit.

S4 References for Supplement

Najbauer, E. E. and Andreas, L. B.: Correcting for magnetic field drift in magic-angle spinning NMR datasets, J. Magn. Reson., 305, 1–4, https://doi.org/10.1016/j.jmr.2019.05.005, 2019.

Press, W. H., Teukolsky, S. A., Vetterling, W. T. and Flannery, B. P.: Minimization or Maximization of Functions, in Numerical Recipes in C: The Art of Scientific Computing, pp. 394–455, Cambridge University Press, Cambridge, 1992.

Římal, V.: Pulse and AU programs for correcting field instabilities in NMR by simultaneous acquisition of a frequency reference (SAFR), ETH Zurich [code], https://doi.org/10.5905/ethz-1007-485, 2022.
